# On the Pathophysiology of Pathological Lying: A Case Report and Literature Review

**DOI:** 10.1192/j.eurpsy.2025.1879

**Published:** 2025-08-26

**Authors:** J. F. Silva, B. P. Brás, C. Machado, A. S. Pinto, A. Lopes

**Affiliations:** 1 Unidade Local de Saúde de Santo António, Porto, Portugal

## Abstract

**Introduction:**

Pathological lying, traditionally known as pseudologia fantastica or mythomania, is characterized by persistent or compulsive lying that often involves elaborate and fantastical narratives. Although the psychiatric community has yet to reach a consensus about its classification as a symptom or as a distinct diagnostic entity, emerging research about its pathophysiology highlights the involvement of complex interactions between neurobiological, psychological, and social factors.

**Objectives:**

The aim of this study is to explore, through a clinical case of an inpatient in whom pathological lying was identified, the possible causes and underlying mechanisms of this condition.

**Methods:**

A case report presentation followed by a non-systematic review of the literature available at PubMed, ScienceDirect and ResearchGate databases, using the MeSH terms “pathological lying” OR “pseudologia fantastica” OR “mythomania”. From a total of 226 abstracts initially screened, we included 51 articles in the final review.

**Results:**

We report the case of a 33-year-old man with a diagnosis of Charcot-Marie-Tooth disease type 2C and comorbid depressive disorder, who was admitted to the emergency department for suicidal ideation. Collateral information from family members was crucial to identify pathological lying, in this case associated with the lack of social relationships, low self-esteem, a desire for autonomy, and poor emotional and behavioral regulation. Although standard blood workup yielded unremarkable findings, imaging studies showed an old lacunar infarction localized to the right hemithalamus. While hospitalized he presented rapid clinical improvement, being discharged with outpatient follow-up. The existing body of evidence on pathological lying fails to capture specific causal factors for this phenomenon. Research into its neurobiological basis examined abnormalities in brain areas responsible for executive functioning, impulse control, and behavioral and emotional responses, such as the prefrontal cortex, the limbic and paralimbic systems, and the right hemithalamus. From a psychological perspective, the pathological liar doesn’t have an external motive for lying; instead, the lie seems to be the purpose in itself, being unconsciously produced to fulfill the need for power and autonomy, to elevate one’s self-esteem, or to repress reality. In addition, certain environmental factors, including childhood trauma, neglect, or abuse, also seem to play a significant role in shaping this type of behavior.

**Conclusions:**

The current research on the pathophysiology of pathological lying is still limited and vague, depicting a multifactorial entity that would benefit from a multidisciplinary approach integrating Psychiatry, Neurology, and Behavioral Sciences. The development of a more comprehensive conceptual model may help practitioners implement formal assessment and management strategies for people suffering from this condition.

**Disclosure of Interest:**

None Declared

